# Blockchain Tree as Solution for Distributed Storage of Personal ID Data and Document Access Control

**DOI:** 10.3390/s20133621

**Published:** 2020-06-27

**Authors:** Sergii Kushch, Yurii Baryshev, Silvio Ranise

**Affiliations:** 1 Department of Electronic and Computer Engineering, University of Limerick, V94 T9PX Limerick, Ireland; 2 Security and Trust Research Unit, Bruno Kessler Foundation, 38123 Povo, Italy; ranise@fbk.eu; 3 Information Protection Department, Vinnytsia National Technical University, 95 Khmelnytske Shose, 21021 Vinnytsia, Ukraine; yuriy.baryshev@vntu.edu.ua

**Keywords:** Blockchain, ID-card, personal data protection, Blockchain Tree, Blockchain for persons’ identification

## Abstract

This paper introduces a new method of Blockchain formation for reliable storage of personal data of ID-card holders. In particular, the model of the information system is presented, the new structure of smart ID-cards and information on these cards are proposed. The new structure of Blockchain, “Blockchain Tree”, allows not only to store information from ID-cards but also to increase the level of security and access control to this information. The proposed Subchains system allows us to integrate Blockchain of the lower level to Blockchain of the higher level, allowing us to create a multilevel protected system.

## 1. Introduction

The use of information resources and government services in the modern world implies unambiguous user identification. To this end, many states create so-called digital personalities. The most famous example of such a system in the EU is Estonia. In this country, every citizen can get not only the usual ID-card for the EU countries, but also a mobile-ID. Using their mobile/digital-ID, an ID-holder may get online access to most government services, remotely open a bank account, register a company, make an appointment for a doctor, etc.

The physical medium of such an identifier is an ID-card [1,2,3,4,5]. All countries of the European Union issue national ID-cards to citizens and residents.

The chip of the card stores information about its owner: full name, gender, national identification number, fingerprints, cryptographic keys, and certificates. A cardholder has the right to use the ID-card as an identity card for travel through the territory of the European Union and for crossing its external borders both for entry and exit from the countries of the European Union and the European Economic Area, including Iceland, Norway, and Switzerland.

We consider the issue of this card’s security, because a single card contains a key to all personal data of a citizen.

Firstly, the chip on a smart card is a sufficiently protected microcomputer that has a microprocessor, a cryptographic coprocessor, and some memory (flash or EEPROM). Unlike a standard microcontroller, access to the memory of a smart card is strictly controlled by the processor. Thus, both reading and recording of the data are regulated by the software of the card itself. Moreover, chip manufacturers are taking measures to prevent unauthorized access (copying all the memory, reprogramming) to the card at electronic and physical levels.

Secondly, all the data in the card is encrypted (in the Estonian map, 2048-bit encryption is used). Thirdly, the downloaded applications (applets) cannot be read from the card by anyone, including the cardholder (an applet can only be erased and a new one should be written instead of the erased one in its place). You can only pass a command to the applet and get a response.

Fourthly, you need a PIN-code for the card, and sometimes two: the first one for authorization, and the second one for confirmation of operations.

However, despite all the advantages of such protection, the system is not secure from unauthorized changes of personal information from the inside (for example, due to server hacking), from creation of fake digital personalities on the basis of which can be used to obtain legal documents and from sale of personal data to external interested organizations (a recent example being Facebook). In addition, the analysis of Internet search results for “buy EU ID-card” shows an abundance of offers to sell clones of real EU documents. Using Blockchain technology [6] can help eliminate the first two threats and increase control over the use of such information by authorized users. Blockchain is a well-known technology used in Bitcoin [7,8,9] and other cryptocurrencies [10,11]. Successful attempts are also being made to introduce this technology in areas of bank transfers [12], logistics [13], energy [14], IoT [15,16], and healthcare [17]. We consider further the advantages and disadvantages Blockchain Technology.

**Advantages**

- It is a decentralized system.
  Since Blockchain data is often stored in thousands of devices on a distributed network of nodes, the system and the data are highly resistant to technical failures and malicious attacks. Each network node is able to replicate and store a copy of the database and, because of this, there is no single point of failure: a single node going offline does not affect the availability or security of the network [18].
- The transparency.
  Each transaction is copied to either computer (node) in the Blockchain network. Every participant can look at all transactions, this also means that each action is showed to participants of the Blockchain. Nobody cannot do anything insensibly [19,20].
- The high security of the Blockchain
  The highly secure nature of the Blockchain technology is achieved on the individual entry into the network because each person who enters the Blockchain is provided with the unique identity that is linked to his/her account. Another reason of the Blockchain security is the reliable chain of the cryptographic hash. When a new block is created, it is necessary to calculate a hash value for the new block. The new hash surely includes the previous hash’s value. This hash is generated automatically by the node key. In this case, it is impossible to change any information in the hash value [19].
- Faster and cost-effective.
  Traditionally, the transaction takes a lot of time in processing and initialing into banking organization. The use of Blockchain technology helps to reduce the time for processing and initialing—from approximately 3 days to several minutes or even seconds [19,20]. This advantage is the same when speaking about using Blockchain in a bank’s system.
- Stability.
  Confirmed blocks are very unlikely to be reversed, meaning that once data has been registered into the Blockchain, it is extremely difficult to remove or change it. This makes Blockchain a great technology for storing critical data where an audit trail is required because every change is tracked and permanently recorded on a distributed and public ledger [18].

**Disadvantages**

- Data modification.
  Another downside of Blockchain systems is that once data has been added to the Blockchain, it is very difficult to modify or delete it. While stability is one of Blockchain’s advantages, it is not always good. For instance, this problem may actually be to fulfill the requirements of GDPR to use the right to oblivion.
- Storage.
  Blockchain ledgers can grow very large over time. The Bitcoin Blockchain currently requires around 200 GB of storage. The current growth in Blockchain size appears to be outstripping the growth in hard drives and the network risks losing nodes if the ledger becomes too large for individuals to download and store [18].
- Human errors.
  As mentioned, Blockchain is immutable, therefore information going into the database needs to be 100% sure and correct; if any mistake happens with data, it cannot be altered [18].
- High power consumption.
  The main disadvantage of the most-used Blockchain consensus algorithm, Proof of Work (POW), is the high energy consumption and, as a result, the high cost of a support Blockchain in general [19].
- The opportunity to split the chain.
  The next problem of the Blockchain is the opportunity to split the chain. The nodes, which are operating to the old software, will not accept the transactions in the new chain. This chain is created with the same history as the chain, which is based on the old software. It is named the fork [19].

Blockchain, by definition, is a distributed database in which each subsequent block containing information is associated with the previous one. The generation of each block must be confirmed by other participants. Nowadays, there are several concepts of the Blockchain consensus algorithms—Proof of Work (POW) [21,22], Proof of Stake (POS) [23,24], Proof of Importance (POI) [25], Proof of Activity (POA) [26], etc. The most common algorithm is Proof of Work. However, while it is well known, this algorithm uses too many resources to generate blocks; it is too slow and in many cases not necessary for building Blockchain. This is why we believe that POW and other resource-intensive algorithms should not be used when it comes to the public and public services; for example, when all nodes of a network are well known and are state organizations. This structure of chain may be used for implementation of Blockchain in systems for checking smart tickets in transport, driver licenses, education degree documents, etc.

There are other known researches performed in the area of person identification utilizing the Blockchain technology. The survey of Blockchain-based identification management performed in [27,28] shows that most projects are focused on utilizing the well-known public Blockchains such as Ethereum or Bitcoin. The main issues of such approach for the considered task are related with the limited resources of public service terminals and especially officials’ card readers, those do not possess enough of both power and computational resources to perform public Blockchain data analysis for the corresponding data. Similar issues appear if mobile devices such as smartphones are used for the purpose. They could be handled by the use of some aggregators for Blockchain data parsing, web servers, or JSON RPCs for instance [27,29], but this way of solution leads to new vulnerability points—the very aggregators themselves. We consider the usage of such aggregators as limiting the Blockchain advantages and drawing them close to ones which do not utilize Blockchain at all and, as such, don not suffer its disadvantages. That is why some of the projects started basing on well-known public Blockchain-based systems, for instance [30], have been migrating to their own Blockchain [31]. However, known works based on their own Blockchains still do not take into consideration gadgets’ limited abilities. Moreover, as it is shown in [32], “pure” Blockchain-governed solutions that do not involve third parties (i.e., officials, public service providers, etc.) into consideration could not be properly implemented for such tasks. The structure of this paper is as follows. In Section 2, we analyze the model of an information system utilizing ID-cards, ID-card structure, and the structure of information in an ID-card. The results obtained in the study are introduced in Section 3. Finally, we present the conclusions obtained from our research and discuss the possibilities for future work in Section 4.

## 2. Background

We further consider the methodology we propose for constructing Blockchain for the network, all nodes of which are verified servers of “Migration Police” that store personal information about citizens and existing ID-cards.

### 2.1. Information System Model

The model of information system utilizing ID-cards-handling consists of the following major entities:

- Citizen ID-cards;
- Data of registered citizens (database in a wide sense);
- Tools for ID-card verification;
- Tools for database administration;
- Tools for new ID-card issuing.

**Figure 1:**
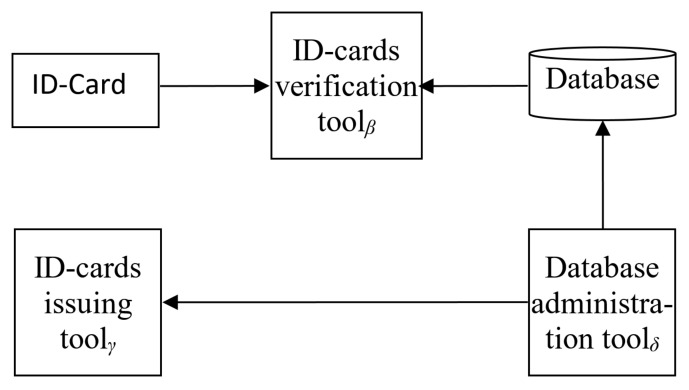
The schematic representation of the ID-card verification and issuing process.

Let us consider the interactions between these entities. All of the entities, but the database, are to be represented by the sets of instances. Let us for the sake of clarity consider just one instance of each of these entities for now: ID-card${}_{\alpha }$, ID-card verification tool${}_{\beta }$, ID-card issuing tool${}_{\gamma }$, and data administration tool${}_{\delta }$. The interaction between them can be presented schematically (Figure 1).

It can be seen from Figure 1 that the entities’ principal interaction occurs during ID-card issuing and ID-card verification. The procedure of ID-card issuing implements the following relations:

$$Issuing=\left\{\begin{array}{c} Accounting:PD\to DB,\\ Manufacturing:PD\to ID, \end{array}\right.$$

where PD is a set of an EU citizen’s personal data that concerns citizenship, DB is a set of database elements used by legal officers during performance of their duties, and ID is a set of all ID-cards issued so far.

It is obligatory for ID and DB to contain corresponding data for a given citizen. The latter is possible only in the case where these sets are isomorphic in relation to contained data DB⇔ID. Consequently, their power should be the same ∥DB∥=∥ID∥ and, for the given i≤∥DB∥,i∈N, the following isomorphic equality is to take place $db_{i}\Leftrightarrow id_{i}$, where $db_{i}\in DB$ and $id_{i}\in ID$. The latter is correct only when DB and ID are both particular instances of sets–lists, i.e., bounded ordered sets.

As mentioned above, the ID-card issuing process is an object of ID-card forgery attack, which can be mathematically described in the following form:

$$\forall pd_{j}\in PD,Manufacturing\left(pd_{j}\right)\notin ID;\exists db_{i}\in DB,\Leftrightarrow Manufacturing\left(pd_{i}\right).$$

This presentation shows that it is essential to have an unattached database entry index to the ID-card, i.e., for the set of η ID-cards, one can add only (η+1)-th element at the given moment of time. Lists could solve this issue, but their ability is not enough, because the given *i*-th element could be replaced. That is why we suggest using data structure, which allows adding information to the DB, but forbids its deletion, replacement, or alteration of Blockchain, where data from the respective ID-card would be bonded to one block.

The other relation shown on Figure 1 is ID-card verification. The process can be presented in the following form:

Verification:ID×DB→{true;false}.

**Figure 2:**
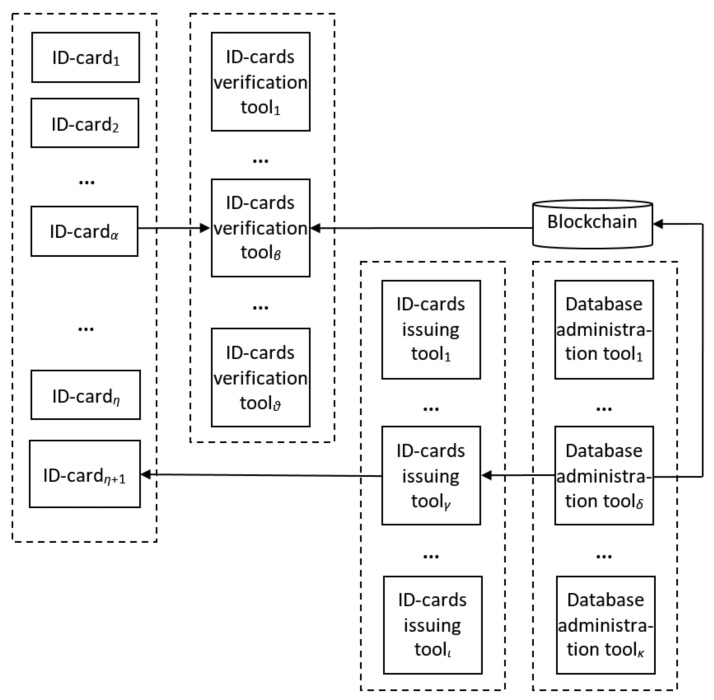
Detailed schematic representation of ID-card verification and issuing process.

In the case when a Blockchain is used for data storage, the verification operator includes block number comparison, in addition to the usual data content and its hash digits comparison. Moreover, fixed Blockchain structure and distribution would help to prevent attacks focused on database content. Bearing in mind the abovementioned results, the scheme presented in Figure 1 could be further detailed by the one shown on Figure 2 for the case of η ID-cards (issuing (η+1)-th one), ϑ existing ID-card verification tools, ι ID-card issuing tools, and κ database administration tools.

It can be seen from Figure 2 that ID-card verification tools and database administration tools are the ones interacting with Blockchain. Therefore, they are to be considered as nodes of the Blockchain. Database administration tools should be considered as so-called full nodes because they are used to add new blocks while the new ID-card issuance process is performed. The peculiarity of the task considered at the article is that just adding new blocks to the conventional chain is not sufficient due to the abovementioned isomorphic relation $db_{i}\Leftrightarrow id_{i}$, so the conventional Blockchain approach is to be modified to reach the goal of the research, while maintaining the Blockchain advantages considered in the introduction.

Similarly to the database administration tools, ID-card verification tools such as public service terminals, customs officials’ and police officers’ card readers. etc., should be considered as so-called light nodes. They should be able to read data from the Blockchain and verify its integrity. Consequently, the tools are able to both perform their “direct” tasks—providing the interface for interaction with the ID-cards, obtaining citizen’s authentication data (by fingerprints for instance), validating ID-cards, etc.—and interact with Blockchain. Therefore, for the case of card readers, they are to be considered as smart-sensors able to support Blockchain interaction protocols. It should be beared in mind that during these protocols’ development, sensors’ computational and energy storage abilities were limited. This is the reason why well-known Blockchains, especially ones utilizing POW consensus algorithm, could not be used for the purposes of this research due to their increased resources demands. Moreover, it is obvious that the main issue to be faced by the card readers, is the difficulty of the related data search at the Blockchain, which should be minimized by the ID-cards and Blockchain architecture.

### 2.2. ID-Card Structure

The ID-cards are used as a citizen authentication factor as well as a tool for Blockchain interaction. The latter is essential in the case when Blockchain is created as shown below in a Section 3.1. In this case, an ID-card user gains additional protection against intrusion into the respective SubBlockchain. For instance, if an intruder tries to fake his identity, he would have to break both country’s security measures and signature, generated by the ID-card. The signature is created by the key derived from user-specific data (e.g., fingerprints) and ID-card-specific data.

To implement this feature, the ID-card should contain a chip that provides interaction with the application interface of the Blockchain. The chip needs to have the following major blocks:

- Encrypted memory, which contains personal data (i.e., respective block or SubBlockchain’s genesis block);
- Private key derivation block—for avoiding private key leakage;
- Signature creation block for validating changes of personal data, which could be performed when certain personal data is changed;
- Interface—for external interaction.

**Figure 3:**
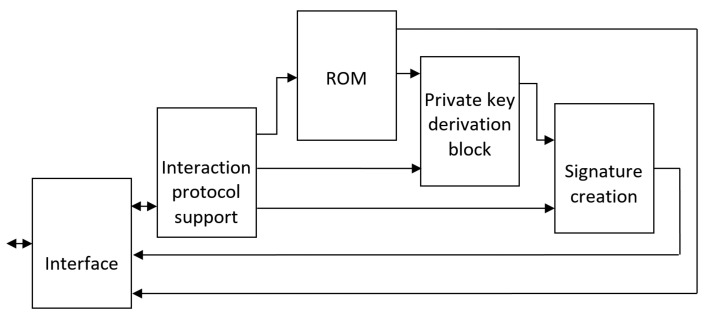
Generalized structure of ID-card processor.

The generalized structure of an ID-card processor is shown in Figure 3.

Interaction with an ID-card could be performed in several ways, so certain protocols should be used. The most common task of an ID-card is providing data. Therefore, in this case, the ID-card would function in the following way:

- Reading interface query and transmitting it to the interaction protocol support block;
- Validating the correctness of the query and determining the data fields of ROM that are to be accessed;
- Sending respective addresses to ROM and transferring them to interface;
- Sending data according to the interface query.

Despite the comparative rarity of the cases where data of the person is changed, in most cases this still should not be performed without this person. To validate such a change, the person must possess the ability to sign such transactions, for instance, by using the card. For this purpose, the following steps should be performed:

- The receiving respective frame-command interaction protocol support block is to wait for additional personal data input;
- After respective data input is received, the interaction protocol support block transfers it to private key derivation block and sends query to respective fields of ROM for ID-card-specific data;
- Using private key as input, the derivation block yields key and sends it to the signature-creation block;
- Meanwhile, interaction protocol support block sends data to be signed to signature-creation block, where the respective signature is obtained and outputted via interface.

### 2.3. Block Structure (Information Structure on a Chip)

The block consists of the following main parts: header, data, block hash. Header structure is determined by Blockchain consensus rules and as such, is heavily dependent on particular protocol implementation. The most common fields of the block headers and the ones used in particular cases are the following:

- Version—contains version of the block, which defines structure of other block’s fields, used consensus protocol peculiarities, presumed forks markers, etc.;
- Hash value of previous block—the integral part of any Blockchain, which provides cryptographic proof of its integrity;
- Hash value of block content—generally used for data integrity protection, but in the considered case it is also essential for rapid Blockchain content search;
- Creation Timestamp—primary intent is to boost protection by limiting potential forger by this field value (its value must be in time window between the blocks preceding and following creation timestamps (Creation Timestamps)), while the field could be used to aid stored data processing;
- Creator identifier of authentication data—is supposed to determine the source of the block (node) because for legal data, all sources, i.e., nodes, are to be determined and authorized by respective government structures.

In this particular case, several sources might act as creators of a transaction. For instance, in case of citizenship changing, the citizen and respective governments are such source. That is why the multisig technique should be used for this type of transaction.

Header may include other metadata depending on the consensus, such as nonce and difficulty for PoW consensus or number of included transactions for Blockchains where this number can vary.

The example of EU ID-card is Estonian ID-card. Table 1 shows the contents of a personal data file stored on an ID-card.

We suggest adding extra areas in the chip memory. Thus, the data part of the block will include the cardholder’s information as it is shown in Table 2.

In order to provide whole block integrity, we suggest using extra hashing of its content, i.e., header and data. The latter hash value is to be used to alter the blocks.

## 3. Main Results

### 3.1. Blockchain for a Database

**Figure 4:**
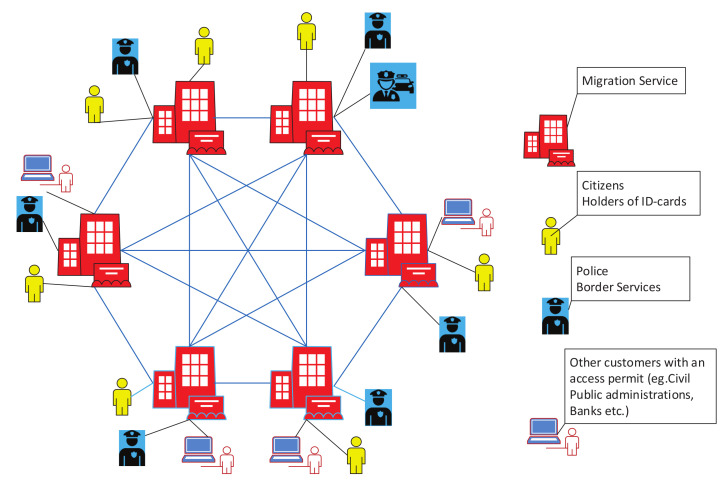
Overall structure of the network. The nodes are police, special services, migration department branches, and other state organizations that have access to personal data. The users of the information are police, migration officers, employees of special services, as well as third parties authorized by the state or holder of the information.

The proposed network structure is a classical peer-to-peer (P2P) topology, in which all elements are interconnected (Figure 4).

**Figure 5:**
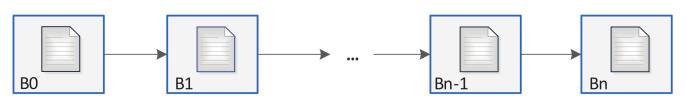
General structure of Blockchain.

The nodes of this network are servers of the regional branches of the migration service, which has the right to issue documents to residents (ID-cards, passports, etc.). All nodes are equal. The network has a fixed number of nodes. Each node is verified and included in the list of approved nodes. This list is stored on each node, and only devices from this list can create new blocks. The structure of the chain has the form shown in Figure 5.

Each block contains information about one ID-card. Transactions are personal data of ID-card holders. Created block must be confirmed by more than 50% + 1 of verified nodes. After that, information is written in the block, added to the chain, and sent to other nodes. Thus, each full node of the system stores a complete Blockchain. In addition, officers’ mobile devices for scanning ID-cards will also serve as Blockchain nodes. However, given the limited resources, these nodes will store the “light” Blockchain—i.e., only block headers—and can connect to the full network as needed. In [15], the authors showed the possibility of creating a Blockchain for a partially connected network. This scheme can also be used in the work of mobile crews (patrols, checkpoints) of public services. In the case of detecting a change in information in an existing block, this block will be automatically replaced with the “right” one that exists on at least 50% + 1 of other nodes of the network.

Given that the blocks in the chain are sequentially linked to each other, the alternation of one block will lead to changing the entire chain. This will be detected and corrected by the rest of the network nodes. Such an algorithm prevents the creation of fake personalities in the system with dates in the past.

### 3.2. Blockchain Integration

It should be beared in mind that the EU is building a common system for recording and controlling issued documents, while building a system for protection of personal data. Therefore, it is necessary to take into account the possibility of integration of the proposed system with a system of a higher level, for example, the EU. We offer two possible options for such integration.

**Figure 6:**
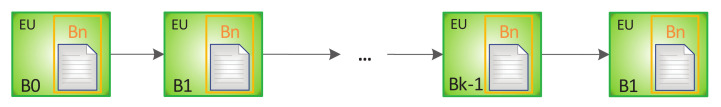
Overall structure of the nested Blockchain.

In the first variant, we take into account that the local Blockchain was created by each country separately and it is necessary to integrate them into a single system. To do this, a possible plan is to create a single EU Blockchain, which will contain blocks of local chains as transactions. In this case, the order of recording specific blocks of each country will be specified separately (Figure 6).

**Figure 7:**
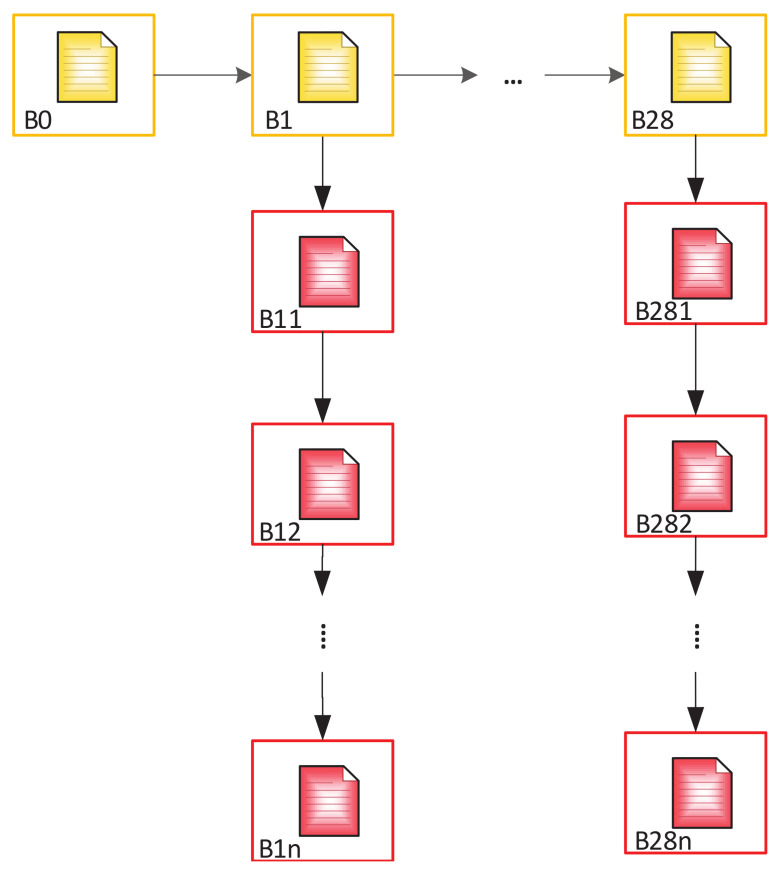
Two-level tree Blockchain for the EU countries.

The second option involves creation of a more complex structure. The main chain consists of 29 blocks: B0—genesis block and 28 (or another number) subsequent blocks (according to the number of EU member countries), each of which is a genesis block for a Subchain, which includes the Blockchain of each member country. This will allow adding new blocks to the main chain when expanding EU in the future. Figure 7 shows the structure of such a Blockchain.

It should be noted that, in the case of the creation of a single system for all EU countries “from scratch”, there will be no need to integrate national systems. It is enough to add a marker of the country that issued the document (the country of creation of the block).

### 3.3. SubBlockchain for Access Control and Evaluation of ID-Cards

**Figure 8:**
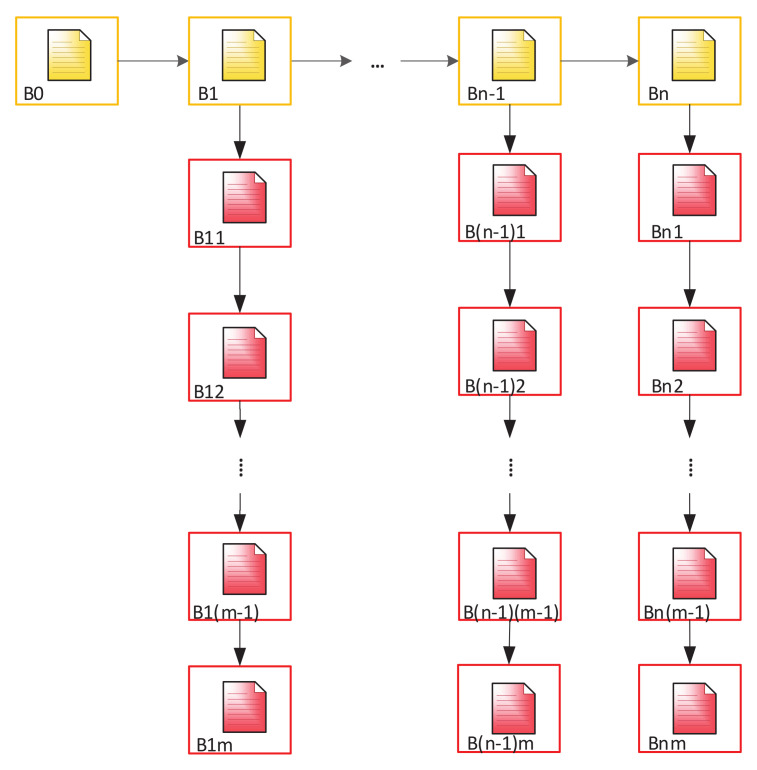
Generalized structure of ID-card processor.

In addition to creating fake personalities, there is also a threat of unauthorized access to personal information as well as its transfer by users to third parties. To control access, it is proposed to create a SubBlockchain, which will record information about each access attempt to a specific block and the use of information from it. The structure of such a circuit is shown in Figure 8.

In this case, the block of the main chain is a genesis block for a Subchain. Thus, we get the number of Subchains equal to the number of blocks in the main chain. Information about access time, device from which the user entered, and information about the user (as well as other necessary items for unambiguous identification of the user) will be recorded in the blocks of this chain.

**Figure 9:**
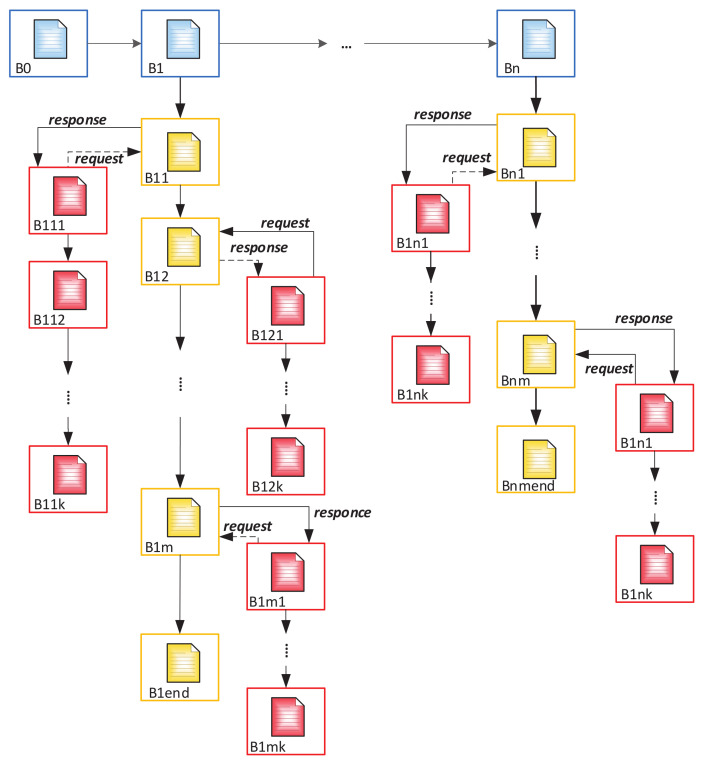
Blockchain Tree. There is a system of three Blockchains that are connected to each other. The blue BC is the main one and contains personal information about the ID-card holder; the yellow BC is a sub-BC and contains information about reissued ID-cards; the red chain is the second sub-BC and contains logs of customers, police, and other officers of special services.

If the national chain is to be integrated into a higher-level system as described above, the scheme for constructing the Subchain has the form shown in Figure 9.

Specialized police scanners and smartphones with an NFC module and/or fingerprint scanner may be used for checking the documents. In this case, the device receives HASH for the checked document from the closest node of the system and compares it with the HASH of the submitted document. Identification of the owner of the document is done by built-in fingerprint sensor.

We will consider in more detail below how the Algorithm 1 works in a possible scenario. There are different possible interactions with our system: onboarding of a new citizen, logging new data, reading an existing data. Let us assume that the user has undergone the procedure for obtaining valid access credentials.
**Algorithm 1:** Algorithm of block formation in a multichain.
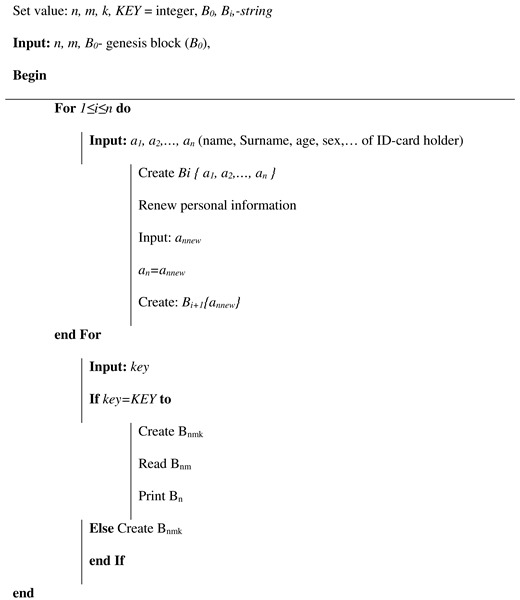


**Figure 10:**
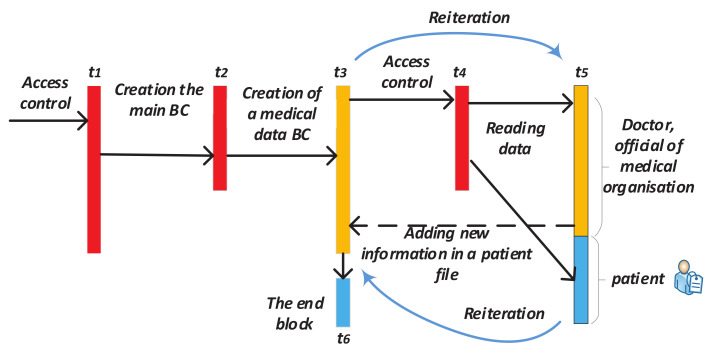
Scheme of possible time steps of the proposed method use for the Blockchain Tree concept.

Thus, the system described above will have the following steps (Figure 10):

1. The appeal of a citizen to authorized department and its registration in it after identity confirmation (before time t1);
2. Creating a block which contains personal information of this person in the main BC (time t1–t2);
3. Creating Block 1 for recording future records history in Subchain 1 (time t2–t3);
4. Creating Block 1 in Subchain 2 for recording access history to Subchain 1;
5. After validation of the cardholder and an official, the blocks are automatically added to Subchains 1 and 2 when the person refers to a government service where it is necessary use ID-cards (time t3);
6. After checking the access right, the citizen’s or an authorized officer’s access to the person’s personal data in Subchain 1 automatically creates a new block in Subchain 2. This block contains information about password holder (in case of unsuccessful entry—about access attempt), date, local time, place, what information was viewed, etc. (time t4–t6);
7. If you need to add new person information, the cycle repeats (new block is created in Subchain 1);
8. Closing Subchain 1 (time t6);
9. After closing Subchain 1, the information can only be viewed (without a chance of adding new data). Subchain 2 continues to grow with each following access or access attempt.

The onboarding of a new person leads to the creation of the following blocks in our Blockchain tree: *(i)* a block in the main Blockchain containing personal information; *(ii)* a genesis block for Subchain 1; and *(iii)* a genesis block for Subchain 2.

Logging of new citizen’s data by an officer leads to creation of a new block in Subchain 1 with automatic creation of the corresponding block in Subchain 2. The block in Subchain 1 contains new data, while the block in Subchain 2 contains log information about officials’ identity, date, local time, place, information that was added, and so on. In the case of unsuccessful entry, the new block contains information about the access attempt.

Instead, the reading of citizen’s data in Subchain 1 automatically creates a new block only in Subchain 2. This block contains information about user’s identity (citizen or official), date, local time, place, information that was viewed, and so on. In case of unsuccessful entry, the new block contains the access attempt.

#### Stop Adding New Records in Blockchain

It is also necessary to envisage a scenario when a person changes the citizenship or dies. In this case, it is necessary to block the possibility to add new blocks to the citizen’ Subchain 1. After Subchain 1 is closed, saved information can only be viewed (without a chance of adding new data). Subchain 2 continues to grow with each following access or access attempt. This will provide additional protection of the system against possible fraud with fake documents. We suggest to add a “Final Block” that marks the end of the Subchain and forbids addition of any new information.

### 3.4. Mathematical Model

Mathematical structure of the main complete chain is

$$F=\overset{\infty }{\underset{i=0}{⋃}}B_{i}.$$

Here, *n*—the modes number of main chain.

Analytic expression, describing the structure of the complete chain (Figure 7), will have the form of Equation (1).

(1) $$F=B_{0}\overset{n}{\underset{i=1}{⋃}}B_{i}\overset{m}{\underset{j=1}{⋃}}B_{j},\left[i,j\in R\right],$$

where *m*—number of elements in a Subchain; *n*—number of elements of the main chain. BC can be described by the following formula—in the case described in Section 3.3 of Figure 8:

(2) $$F=B_{0}\overset{n}{\underset{i=1}{⋃}}B_{i}\overset{m}{\underset{j=1}{⋀}}B_{j},\left[i,j\in R\right].$$

In the case of creating an additional Subchain for the control of personal data, Equation (3) will take the following form:

(3) $$F=B_{0}\overset{n}{\underset{i=1}{⋃}}B_{i}\overset{m}{\underset{j=1}{⋀}}B_{j}\overset{p}{\underset{j,k}{⋃}}B_{j,k},\left[i,j,k\in R\right]$$

The BC structure described in Figure 6 can be represented as follows:

(4) $$F=B_{0}\overset{28}{\underset{i=1}{⋃}}B_{i}\overset{m}{\underset{j=1}{⋃}}B_{i,j},\left[i,j\in R\right],$$

where $B_{0},B_{i}$—the blocks which constitute the main chain (EU chain); $B_{j}$—the blocks which constitute the chains of each member-country.

Then, taking into account the second Subchain storing information about access to personal data:

(5) $$F=B_{0}\overset{28}{\underset{i=1}{⋃}}B_{i}\overset{m}{\underset{j=1}{⋃}}B_{i,j}\overset{p}{\underset{k=1}{⋃}}B_{i,j,k},\left[i,j,k\in R\right].$$

## 4. Summary and Discussion

In this paper, we introduce a novel methodology based on Blockchain for building storage, access control, and document verification mechanisms for migration control area. The proposed work is based on Subchains, connected to a main Blockchain and each other. The solution is more secure than those that exist currently due to the use of mutual intersections of several Blockchains in the one system. This makes the process of hacking and falsification of critical information more difficult since, in the event of an attack, it will be necessary to change not one but several Blockchains, which considerably increases the cost of such an attack and makes it unprofitable for the attacker.

The noted above methodology for building a storage system, access control, and document verification can be used not only for ID-cards but also for other documents, such as driver’s licenses, education documents, personal medical information, social security cards, etc.

The proposed methodology still requires further improvement in order to contribute to its reliable implementation and legal compliance (in particular, referring to GDPR). For example, we left out some problems of building real networks, such as devices and communication line delays. In our future work, we will consider the problem of using various consensus algorithms with different types of Blockchains. Furthermore, this work did not address the use of specialized equipment for checking documents in the field with the creation of a separate closed network and using these devices as nodes of such a network. These questions will be considered and presented in the next paper.

## Figures and Tables

**Table 1:** Contents of a personal data file stored on an ID-card.

N	Content	Example	Length (Bits)
1	Surname	Smith	Max 28
2	First name line 1	John	Max 15
3	First name line 2		Max 15
4	Sex	M	1
5	Nationality	POL	3
6	Date of birth	01.01.1971	10
7	Personal ID code	37101010021	11
8	Document number	X0010536	8 or 9
9	Expiry date	13.08.2019	10
10	Place of birth	POOL/POL	Max 35
11	Date of issuance	13.08.2014	10
12	Permit type	PERMANENT	Max 50
13	Notes line 1	EL KODANIK/EU CITIZEN	Max 50
14	Notes line 2	ALALINE ELAMISOIGUS	Max 50
15	Notes line 3	PERMANENT RIGHT OF RESIDENCE	Max 50
16	Notes line 4	LUBATUD TOOTAD	Max 50

**Table 2:** Contents of personal data stored on an ID-card.

N	Content	Example	Length (Bits)
1	Community of issue	Milano	Max 28
2	Serial number	AA00000BB	Max 15
3	First name line 1	John	Max 15
4	First name line 2		Max 15
5	Surname	Smith	Max 15
6	Place of birth	Milano	Max 35
7	Sex	M	1
8	Nationality	ITA	3
9	Date of birth	01.01.1971	10
10	Stature	186	3
11	Citizenship	ITA	3
12	Image of the holder’s signature	“digital scan”	50
13	Validity for expatriation	13.08.2014	10
14	Photography	“Digital photo”	Max 50
15	Images of 2 fingerprints (one finger of the right hand and one finger of the left hand)	“Digital photo”	Max 50
16	Parents (in the case of a minor’s card)		Max 50
17	Fiscal Code (Personal ID code)	ABCDEF00B00A111W	16
18	Address of residence	Verona str., 1, Rome	Max 50
19	Tax code in the form of a barcode	Barcode	Max 100
20	Information about the documents on the basis of which the ID-card was issued	-	Max 50
21	The number of the block storing information about the previous ID-card (in case of renewal or update of the card). This parameter = 0 if the card is being issued for the first time	-	Max 50
22	ID of migration police officer who created personal file of a citizen	-	Max 50
23	Notes line 4	Permanent residence	Max 50
24	Notes line 4	-	Max 50
25	Notes line 4	-	Max 50
